# Management Strategies in Advanced Uterine Leiomyosarcoma: Focus on Trabectedin

**DOI:** 10.1155/2015/704124

**Published:** 2015-05-18

**Authors:** Frédéric Amant, Domenica Lorusso, Alexander Mustea, Florence Duffaud, Patricia Pautier

**Affiliations:** ^1^Department of Obstetrics and Gynecology, UZ Gasthuisberg, Katholieke Universiteit Leuven, Herestraat 49, Box 7003, 3000 Leuven, Belgium; ^2^Gynecologic Oncology Unit, Fondazione IRCCS National Cancer Institute, Via Venezian 1, 20133 Milan, Italy; ^3^Department of Gynecology and Obstetrics, University Hospital Greifswald, Ferdinand-Sauerbruch-Strasse, 17475 Greifswald, Germany; ^4^Department of Medical Oncology, La Timone University Hospital, 264 rue Saint Pierre, 13385 Marseille, France; ^5^Département de Medecine, Institut Gustave-Roussy, 114 rue Edouard Vaillant, 94805 Villejuif Cedex, France

## Abstract

The treatment of advanced uterine leiomyosarcomas (U-LMS) represents a considerable challenge. Radiological diagnosis prior to hysterectomy is difficult, with the diagnosis frequently made postoperatively. Whilst a total abdominal hysterectomy is the cornerstone of management of early disease, the role of routine adjuvant pelvic radiotherapy and adjuvant chemotherapy is less clear, since they may improve local tumor control in high risk patients but are not associated with an overall survival benefit. For recurrent or disseminated U-LMS, cytotoxic chemotherapy remains the mainstay of treatment. There have been few active chemotherapy drugs approved for advanced disease, although newer drugs such as trabectedin with its pleiotropic mechanism of actions represent an important addition to the standard front-line systemic therapy with doxorubicin and ifosfamide. In this review, we outline the therapeutic potential and in particular the emerging evidence-based strategy of therapy with trabectedin in patients with advanced U-LMS.

## 1. Introduction

Uterine leiomyosarcomas (U-LMS) are a group of rare and aggressive mesenchymal tumors, which comprise ~1% of all uterine malignancies and a third of uterine sarcomas [1, 2]. The incidence of U-LMS is about 0.55 cases per 100,000 women per year [3]. The diagnosis of uterine sarcomas is frequently discovered incidentally on histopathology review following hysterectomy. The most common uterine tumor, endometrial cancer, originates from the endometrial lining and results in early bleeding in its development. Therefore, early diagnosis is common since endometrial sampling yields malignant cells. In contrast, endometrial sampling for early U-LMS is likely to be negative and endometrial involvement resulting in vaginal bleeding only occurs when the tumor has reached a certain volume. In addition, for most cases, a confirmatory diagnosis cannot be made preoperatively, since there are no simple objective imaging characteristics that can objectively distinguish between benign and malignant mesenchymal growths [4]. Diagnosis of U-LMS commonly signifies an aggressive clinical course with a predilection for early hematogenous spread and development of lung metastases within two years of primary therapy [5]. Additionally, the metastatic recurrence rate even in patients diagnosed with localized early stage disease exceeds 50% according to the International Federation of Gynecology and Obstetrics (FIGO) [6, 7]. Therefore, optimal management of U-LMS is challenging and typically involves a multidisciplinary team whose approach generally depends on the disease spread (i.e., localized versus disseminated disease). Complete surgical resection is the mainstay of treatment for localized U-LMS. Indeed, the absence of primary surgery [5] or incomplete cytoreduction [8] predicts poor survival. An “en-bloc” resection is highly recommended for U-LMS as morcellation of the tumor or uterus in total increases the rate of the abdominopelvic dissemination causing an iatrogenically advanced stage disease that translates to a worsened progression-free survival (PFS) and overall survival (OS) [9]. Usually, total abdominal hysterectomy (including removal of the cervix) with or without bilateral salpingoophorectomy (BSO) is performed [5, 10]. Noteworthily the incidence of occult ovarian (<4%) and lymph node metastases (<3%) in U-LMS is very low and is most commonly associated with extrauterine disease [11–14]. A large retrospective population study failed to demonstrate both a statistical difference in the 5-year disease-specific survival (DSS) for women who did or did not undergo BSO at the time of hysterectomy and comparable median OS for women who underwent or not lymphadenectomy [5]. Therefore, ovarian-sparing surgery may be considered in premenopausal women with early stage disease, while lymph node dissection should be reserved only for patients with clinically suspicious and enlarged lymph nodes without compromising outcome [5, 13, 14]. In metastatic disease the role of surgery and locally ablative therapies depends upon the patients' age and general condition, extent of disease, and the aims of treatment. Optimal metastasectomy, either pulmonary or extrapulmonary, has become a standard intervention in carefully selected patients [15, 16]. Pulmonary metastasectomy is the most widely studied and has been associated with 5-year survival rates ranging from 25% to 53% [16–19]. Patients with isolated, unilateral, or limited metastases, an excellent performance status, and a relatively prolonged disease-free interval may be considered as suitable candidates for metastasectomy. The principal predictors of improved outcome following metastasectomy include optimal complete resection of all detected lesions without significant surgical morbidity and prolonged time to first recurrence (>12 months) [15, 16].

Several retrospective, nonrandomized studies had suggested an improved local control without demonstrating a significant survival benefit in patients with resected U-LMS treated with adjuvant pelvic radiotherapy [20, 21]. To overcome the limitations of retrospective noncomparative studies, the European Organization for Research and Treatment of Cancer-Gynecological Cancer Group (EORTC-GCG) conducted the only prospective, randomized phase III study of radiotherapy in uterine sarcomas, comparing adjuvant pelvic radiation (51 Gy in 28 fractions over five weeks) with observation [22]. This study was opened in 1988 and ran over a 13-year period to accrue a total of 224 patients with completely resected FIGO Stage I and II uterine sarcomas, including 103 patients with U-LMS. For those with U-LMS there was no benefit for radiotherapy for either disease-free survival or OS. Based on these findings, the authors concluded that there was no evidence for the routine use of postoperative pelvic radiotherapy. Those results have been reinforced by the results of a much larger population-based study using the Surveillance, Epidemiology, and End Results (SEER) database that reported on outcomes of 1396 women treated for U-LMS, of whom 310 (23%) had undergone adjuvant postoperative radiotherapy, which reported that the addition of radiotherapy had no impact on 5-year DSS [5].

Similarly there is no good evidence for the routine use of adjuvant chemotherapy since all data to date have not conclusively proven adjuvant chemotherapy to be of clear benefit for patients with localized resectable disease [23–25]. A large meta-analysis of 14 studies of doxorubicin-based adjuvant chemotherapy for localized resectable soft tissue sarcoma (STS) in adults included 1568 patients of whom 264 had uterine sarcoma. Even though adjuvant chemotherapy appeared to significantly improve time to local and distant recurrence and overall recurrence-free survival, it had no significant impact on OS [26, 27]. Recently, the EORTC sarcoma group carried out the largest prospective randomized study of adjuvant chemotherapy in STS with the aim of finally answering the question of usefulness of such an approach [28]. In that study 351 patients with completely resected high grade STS were randomly assigned to receive either adjuvant chemotherapy (doxorubicin and ifosfamide with lenograstim) or no chemotherapy (control group). No benefit for adjuvant chemotherapy was found between groups neither for relapse-free survival nor for median OS and 5-year OS rate (chemotherapy group: 66.5% versus observation control group: 67.8%).

## 2. Treatment of Advanced Uterine Leiomyosarcoma

### 2.1. Chemotherapy

Despite adequate surgical resection of U-LMS, even in early stage, patients remain at high risk for local and distant recurrence [29]. Optimal treatment of advanced or unresectable disease generally involves palliative systemic chemotherapy regimens with poor prognosis demonstrating a median PFS of ~5 months and median OS of ~12 months, and 5-year DSS rates of less than 30% [5, 30]. In STS generally and in U-LMS specifically, chemotherapeutic options that achieve sustained responses remain limited [31]. Standard first-line chemotherapy has been largely unchanged for three decades and remains doxorubicin with or without ifosfamide (Table 1) [32–35]. Doxorubicin monotherapy has consistently demonstrated an objective response rate (ORR) of approximately 13–25% with response duration typically lasting less than 6 months and median OS of ~12 months [34, 35], whereas ifosfamide alone had a response rate of 17.2% with a median response duration of 3.8 months and median OS of 6 months [36]. Indeed, the use of doxorubicin (25–50 mg/m^2^) in combination with ifosfamide (5–10 g/m^2^) chemotherapy has resulted in higher ORR (doxorubicin 50 mg/m^2^ and ifosfamide 5 g/m^2^ showed an ORR of 30.3%; doxorubicin 75 mg/m^2^ and ifosfamide 10 g/m^2^ showed an ORR of 48%), but this has been at the expense of increased treatment-related toxicities due to overlapping myelotoxicity and worsening in patients' quality of life and with no impact on OS (Table 1) [32, 37]. An open-label randomized phase II study that evaluated the efficacy of sequential high-dose doxorubicin and ifosfamide compared with standard-dose doxorubicin showed no advantage to sequentially adding ifosfamide to doxorubicin as compared to doxorubicin alone in first-line treatment of advanced STS [38]. In that study patients were randomly assigned to either doxorubicin 75 mg/m^2^ given as a bolus injection every three weeks (q3w) for six cycles (standard arm) or high-dose doxorubicin at 30 mg/m^2^ per day for three days every two weeks for three cycles followed by ifosfamide at 12.5 g/m^2^ as a continuous 5-day infusion, once q3w for three cycles with filgrastim or pegfilgrastim support. The ORR was 24.1% and 23.4% in the high-dose and the standard doxorubicin arm, respectively, and median PFS was shorter in the high-dose arm (24 weeks) compared with the standard arm (26 weeks). Febrile neutropenia (23% versus 7%) and study discontinuation due to drug-related toxicity (11% versus 1%) were more common in the high-dose sequential arm [38]. Recently, the results of a randomized, controlled phase III EORTC 62012 trial demonstrated that the combination of doxorubicin 75 mg/m^2^ and ifosfamide 10 g/m^2^ as first-line therapy for patients with advanced or metastatic STS (*n* = 445) failed to significantly improve OS (median OS: 14.3 months versus 12.8 months; *p* = 0.076) and was considerably more toxic than doxorubicin 75 mg/m^2^ alone [39]. Moreover, all grade 3/4 toxicities were more common with doxorubicin and ifosfamide than with doxorubicin alone (leucopenia 43% versus 40%, neutropenia 42% versus 37%, febrile neutropenia 46% versus 13%, anemia 35% versus 5%, and thrombocytopenia 33% versus <1%).

Few chemotherapy agents or combinations have been demonstrated to be active in U-LMS that has progressed after doxorubicin-based treatment. A Gynecologic Oncology Group (GOG) phase II trial evaluated the antitumor activity and toxicity profile of gemcitabine (gemcitabine 1000 mg/m^2^ on days 1, 8, and 15 of a 4-week cycle) as second-line chemotherapy in patients with recurrent or persistent U-LMS [40]. The schedule was well tolerated and an ORR of 20.5% (2.3% complete response and 18.2% partial response) was observed among 42 evaluable patients with the median duration of 4.9 months. In addition, seven (15.9%) patients achieved stable disease (SD). The combination of gemcitabine and docetaxel has recently emerged as a promising treatment for U-LMS, thus representing a valuable addition to doxorubicin and ifosfamide in the treatment of metastatic U-LMS (Table 1) [41]. In three prospective phase II studies the combination of gemcitabine and docetaxel has demonstrated efficacy as first- or second-line therapy for advanced U-LMS associated with a high ORR ranging from 27% to 53%, median PFS from 4.4 to 6.7 months, and median OS from 14.7 to 17.9 months [42–44]. However, for the combination of gemcitabine plus docetaxel as the second-line therapy, 50% of patients received red blood cell transfusions, 13% received platelet transfusion, and 8% of patients had pulmonary toxicity [42]. Similarly, in a randomized trial in patients with metastatic STS of multiple histologies, the combination of gemcitabine and docetaxel yielded superior ORR (16 versus 8%), median PFS (6.2 versus 3.0 months, *p* = 0.02), and median OS (17.9 versus 11.5 months, *p* = 0.03) to gemcitabine alone, but with increased toxicity [45]. Unfortunately, these encouraging efficacy results could not be confirmed in a subsequent French trial that included 133 patients with advanced STS as that observed an overall response with gemcitabine plus docetaxel combination of 18.4% and with no statistical difference between leiomyosarcomas and other histological subtypes (24.2% versus 10.4%; *p* = 0.06) [46]. The French Sarcoma Group recently completed a randomized multicenter phase II TAXOGEM study that aimed to evaluate the efficacy and toxicity of single-agent gemcitabine versus gemcitabine plus docetaxel as second-line therapy in patients with metastatic or unresectable uterine and nonuterine LMS [47]. A total of 90 patients (46 with U-LMS) received either single-agent gemcitabine (gemcitabine 1000 mg/m^2^ on days 1, 8, and 15 of a 4-week cycle) or a combination of gemcitabine and docetaxel (gemcitabine 900 mg/m^2^ i.v. on days 1 and 8, plus docetaxel 100 mg/m^2^ i.v. for one hour on day 8 of a 3-week cycle with lenograstim). This study failed to show the superiority of gemcitabine plus docetaxel over gemcitabine alone since single-agent gemcitabine (ORR; 19%; median PFS: 5.5 months) yielded results similar to those of gemcitabine plus docetaxel (ORR: 24%; median PFS: 4.7 months) in this trial, but with less toxicity (one toxic death occurred in the gemcitabine plus docetaxel arm) [47]. In addition, the results of an analysis that pooled individual data from 12 patients with U-LMS from the SARC002 randomized phase II study and 40 patients from TAXOGEM study also showed no statistical difference between gemcitabine (ORR: 18%; median PFS: 4.9 months) and gemcitabine plus docetaxel (ORR: 23%; median PFS: 6 months) as mixed-line therapy (second-line therapy for >77% of patients) [48]. Therefore, the use of the combination of gemcitabine and docetaxel in U-LMS still remains controversial.

The preliminary results of a phase II prospective study of combination therapy with carboplatin and pegylated liposomal doxorubicin (PLD) in 40 patients with advanced or recurrent gynecologic sarcomas (14 with U-LMS) reported a high ORR and disease control rate (DCR; ORR plus SD) of the combination (ORR = 33.3%; DCR = 70.4%) and a 12-month PFS and OS rates of 32.5% and 77.0%, respectively, with the favorable safety profile [58]. A variety of other cytotoxic agents, including temozolomide [59–61], topotecan [62], thalidomide [59], paclitaxel [63], and cisplatin [64], have demonstrated very modest activity in U-LMS.

### 2.2. Other Approaches: Targeted and Hormonal Therapy

#### 2.2.1. Targeted Agents

Sarcomas are vascular tumors with higher levels of vascular endothelial growth factor (VEGF) expression than most other solid tumors and this provides a potential target that could be exploited through inhibition of angiogenesis [10]. To date the only approved targeted therapy for patients with metastatic nonadipocytic STS after previous chemotherapy is pazopanib hydrochloride, a multitargeted tyrosine kinase inhibitor, including VEGF-1, VEGF-2, and VEGF-3. In 2012, the European Medicines Agency and the U.S. Food and Drug Administration (FDA) have approved pazopanib based on the results of the pivotal, randomized, double-blind, placebo controlled, multicenter, phase III PALETTE study in 369 patients (165 with U-LMS), in which pazopanib significantly increased the time that patients remained progression-free compared with placebo (median PFS: 4.6 versus 1.6 months; *p* < 0.001) [65]. The 3-month improvement in PFS was observed despite only a 6% ORR in the pazopanib group, suggesting that the majority of patients benefited in the form of SD. However, the protocol-specified final analysis of OS showed that longer PFS with pazopanib did not translate into an improvement in OS (median OS: 12.5 versus 10.7 months; *p* = 0.25).

The role of bevacizumab, a monoclonal antibody directed against VEGF, in addition to fixed-dose-rate gemcitabine plus docetaxel (GD), has also been investigated as first-line treatment for metastatic U-LMS in a phase III, double-blind, placebo-controlled trial [66]. In that study 102 patients were randomly assigned to either gemcitabine (900 mg/m^2^)/docetaxel (75 mg/m^2^) plus bevacizumab (B; 15 mg/kg; *n* = 50) or GD plus placebo (P; *n* = 52). Unfortunately, the addition of bevacizumab to the combination of GD failed to improve PFS (GD + B: 4.1 months versus GD + P: 6.2 months), OS (GD + B: 23.3 months versus GD + P: 19.4 months), or ORR (GD + B: 32% versus GD + P: 36%) and worsened the overall toxicity profile. Formerly, a phase Ib study of the combination of docetaxel, gemcitabine, and bevacizumab in chemotherapy-naïve patients with advanced or recurrent STS reported the ORR of 31.4%, with five complete and six partial responses, and an additional 18 had SD lasting for a median of 6 months, similar to historical response rates with this cytotoxic combination alone [67]. Nevertheless, some concerning adverse events were attributed to bevacizumab as one patient died of a pulmonary embolism following surgery for a bowel perforation, one patient developed a grade 3 wound dehiscence, and another experienced a grade 3 tumor-related hemorrhage. Additionally, in a phase II study, the antitumor activity and tolerability of bevacizumab and doxorubicin were evaluated in 17 patients with metastatic STS (seven had U-LMS) who received up to one nonanthracycline prior therapy [68]. The ORR was lower than might be expected with single-agent doxorubicin in U-LMS, as there were only two partial responses (12%) and 11 disease stabilizations (65%). Of major concern, despite careful monitoring and the standard use of dexrazoxane, was the unexpected cardiac toxicity with the combination with a 35% incidence of grade 2 or worse cardiotoxicity.

Two other multitargeted protein tyrosine kinase inhibitors with activity against multiple VEGF isoforms, sunitinib and sorafenib, have also been evaluated in U-LMS with disappointing results as neither has met prespecified criteria to warrant further clinical development [69, 70]. Currently, an ongoing EORTC randomized double-blind phase II study (ClinicalTrials.gov Identifier: NCT01979393) evaluates the role of maintenance therapy with cabozantinib (XL184), an oral tyrosine kinase inhibitor, in high-grade undifferentiated uterine sarcoma (HGUS) following surgery and stabilization or response to doxorubicin ± ifosfamide or in patients with metastatic (HGUS) as first-line treatment.

#### 2.2.2. Hormone Therapy

To date, the exact role of hormonal therapies in U-LMS is poorly defined despite some hints of efficacy due to lack of prospective validation with a control arm. The immunohistochemical expression of estrogen (ER) receptors (40–100%) and progesterone receptors in U-LMS (17–100%) is of relevance as it provides a possible therapeutic strategy for treatment [71–76]. It has been reported that hormone receptor positivity may have prognostic implications, with some studies relating hormonal expression to improved PFS and OS, particularly in cases with disease confined to the uterine body [72, 75, 77]. For instance, in a subset of patients with recurrent U-LMS with an indolent evolution, with a disease-free interval of ≥6 months, it is more likely to express hormonal receptors that may allow targeted treatment. Therefore, for those highly selected patients, with a less aggressive growth pattern, hormonal treatment or metastasectomy may be considered rather than a new line of chemotherapy [78, 79]. In a recent retrospective study of 54 patients (34 were ER positive) with uterine sarcoma they demonstrated improved OS when compared with ER negative patients (median OS: 36 versus 16 months, *p* = 0.004) [75]. On multivariate analysis, ER positivity retained significance as an independent predictor of survival, after controlling for stage, age, histology, and the use of pelvic radiotherapy (*p* = 0.03). Another retrospective study of patients with advanced or recurrent U-LMS treated with an aromatase inhibitor included 34 patients with measurable disease [76]. Best objective response was partial response in three patients (9%), all of whom were ER positive, and SD occurred in a further 11 (32%) patients. The median PFS was 2.9 months (95% confidence interval (CI): 1.8–5.1 months), with superior PFS rates for ER and progesterone-positive tumors as compared with patients whose tumors did not express hormone receptors who did not derive any benefit. While this study provides some evidence of efficacy, this data must be interpreted with caution since, in the absence of a no-treatment control group, the prolonged PFS cannot be attributed solely to the activity of the aromatase inhibitor treatment in this retrospective highly selected group of patients [76]. Therefore, prospective validation with a control arm is required. Only one prospective phase II study of aromatase inhibition with letrozole in estrogen and/or progesterone receptor-positive U-LMS has been reported [80]. The primary endpoint was the PFS at 12 weeks. Among 27 patients enrolled, no objective responses were observed and the best response was SD in 14 patients, but it reported a 12-week PFS rate of 50% with a median duration of treatment of 2.2 months. Overall, progestins and aromatase inhibitors seem to be a reasonable option in patients with estrogen receptor/progesterone receptor-positive, small volume, and/or slowly progressive disease and for whom neither resection nor cytotoxic chemotherapy is warranted.

### 2.3. Treatment Endpoints and Response Assessment in Advanced Uterine Leiomyosarcoma

The optimal treatment for women with U-LMS is developing in parallel with our understanding of the pathways and networks controlling tumorigenesis, cell signaling, proliferation, and cell death. However, decision-making strategies for optimal treatment of U-LMS are complex as the difficulty lies in knowing where new drugs or treatment regimens, such as monotherapy or combination, fit in the treatment algorithm. This also represents challenges in setting treatment expectations, optimal timing, and sequencing, particularly in the development of new clinical trials. The most controversial issue of the STS treatment in general surrounds the phenomenon of the observed clinical benefit in absence of objective response that has potentially important implications for the design of future studies [81, 82]. The inappropriateness of ORR according to the Response Evaluation Criteria in Solid Tumors (RECIST) criteria as a surrogate of clinical benefit appears to be particularly relevant in STS, since it has been shown that patients with STS may derive therapeutic benefit in the absence of tumor shrinkage qualifying for complete or partial response [83]. Therefore, the selection of clinically meaningful objectives and standardized study endpoints is critical. Now it seems largely recognized that disease stability and PFS are more relevant endpoints in STS than ORR [82]. PFS, a time-to-event endpoint that captures benefit from prolonged responses and disease stabilization, has become accepted as the most useful endpoint of efficacy in phase II studies in STS [84]. With this in mind, the EORTC, in an analysis of a large database of clinical trials with standard agents and various experimental drugs, has established 3- and 6-month PFS rates of at least 39% and 14%, respectively, as the thresholds criteria to define drug activity in pretreated STS [82]. Importantly, the occurrence of progression is the main cause of drug discontinuation in clinical practice and clinical studies. From the clinical perspective, the most important issue is not to discontinue the treatment on the basis of standardized assessment of tumor response for treatments which may have an atypical pattern of response, such as delayed responses to trabectedin in which shrinkage was not initially detected or even appeared after tumor increase [52]. This further underlines the importance of correct definition and interpretation of tumor progression in the decision-making strategy for treatment discontinuation. Upcoming research may also consider some new endpoints such as assessment based on density using contrast-enhancement sequences according to Choi assessment [85, 86] and the use of ^18^fluorodeoxyglucose- (FDG-) positron emission tomography (PET-CT) imaging in assessing response to trabectedin treatment [87, 88], as well as evaluation of clinical or symptomatic benefit, which includes time to progression, the growth modulation index (GMI), progression arrest rate, and health-related QoL [89]. In particular, tumor assessment based on Choi criteria seems to be a useful tool for evaluation of response to trabectedin since atypical radiological patterns of response, such as massive central tumor necrosis or tumor calcification, associated with clinical improvement have been previously reported [90, 91].

## 3. Trabectedin

Trabectedin (Yondelis) is a tetrahydroisoquinoline alkaloid, originally isolated from the marine tunicate* Ecteinascidia turbinata* and currently produced synthetically. Trabectedin has a unique mechanism of action based on interaction with the minor groove of the DNA double helix, which triggers a cascade of events that interfere with several transcription factors, DNA binding proteins, and DNA repair pathways, resulting in G2-M cell cycle arrest and ultimately apoptosis [92]. Trabectedin cytotoxicity is influenced by the functional nucleotide excision repair (NER) and deficient homologous recombination repair (HRR) machinery [93]. Consequently, trabectedin shows decreased activity (from 2- to 8-fold) in NER-deficient cell lines, while cells deficient in HRR are approximately 100 times more sensitive to the drug, indicating that trabectedin causes DNA double-strand breaks [93–97].

Nevertheless, emerging evidence indicates that trabectedin has pleiotropic mechanisms of action, since, in addition to inducing direct growth inhibition, cell death, and differentiation of malignant cells, trabectedin at therapeutic concentrations has selective immunomodulatory properties as a result of the inhibition of production of factors that promote tumor growth, progression, and the inhibition of tumor-promoted angiogenesis [92]. Data suggest that trabectedin selectively targets monocytes and tumor associated macrophages (TAMs) and downregulates the production of inflammatory mediators, which induces changes in the tumor microenvironment contributing to its antitumor activity [92, 98–100]. The markedly reduced production of proinflammatory mediators, such as CCL2, interleukin-6 (IL-6), and the proangiogenic VEGF, may underlie the strong association between chronic inflammation and cancer progression [98–101]. Taken together, trabectedin is more than a cytotoxic drug given that it also has immunomodulatory and antiangiogenic properties which potentially contribute to a delayed response with a prolonged stabilization [102]. Consequently, the characteristic late and long-lasting responses reported with trabectedin have now gained greater theoretical support from the perspective of considering trabectedin as a multitarget drug with far more multifaceted activity than originally formulated [103, 104]. This is an active area of research both in preclinical and translational settings.

### 3.1. Trabectedin in Soft Tissue Sarcoma

The efficacy of trabectedin as salvage chemotherapy in adults with advanced, recurrent STS was assessed in three nonrandomized phase II trials [53, 54, 56] and in chemotherapy-naïve patients with unresectable advanced STS of multiple histologies [55]. A phase II randomized registration ET-743-STS-201 study (ClinicalTrials.gov Identifier: NCT00060944) in 270 patients with advanced liposarcoma (*n* = 93, 34.4%) and leiomyosarcoma (*n* = 177, 65.6%; 30 patients, 17% with U-LMS) after failure of prior conventional chemotherapy demonstrated a superior disease control of trabectedin 1.5 mg/m^2^ given as a 24-hour i.v. infusion q3w compared with a weekly trabectedin regimen (0.58 mg/m^2^; 3-hour i.v. infusion for three consecutive weeks in a 4-week cycle) in terms of longer time to progression (median TTP: 3.7 versus 2.3 months; *p* = 0.0302), median PFS (3.3 versus 2.3 months; *p* = 0.0418), and median OS (13.9 versus 11.8 months; *p* = 0.1920) [57]. These benefits from trabectedin therapy in patients treated using a 24 h infusion q3w were highlighted by PFS rate at 3 months (51.5%) and 6 months (35.5%), which surpassed the thresholds criteria established by the EORTC to define drug activity in pretreated STS [82]. Based on these results, in 2007, trabectedin was the first anticancer marine-derived drug to be approved in the European Union and in many other countries worldwide for the treatment of adult patients with advanced STS after failure of anthracyclines and ifosfamide or for those patients who are unsuitable to receive these agents [105].

Although the response rate to trabectedin in pretreated patients with STS is rather low (<18%), this drug has demonstrated prolonged disease control, with a DCR of 50–60%, and large median OS time that exceeds 12 months [15–18] with major benefits in liposarcoma and leiomyosarcoma compared to other STSs. Noteworthily delayed responses compared with other agents are observed with trabectedin (median time to observe an ORR = 5.3 months), which may account for the differences in clinical benefit, since an early and prolonged administration of trabectedin appears to be associated with improved efficacy outcomes when compared with short-term and later treatments [52, 53, 106]. Recent evidence have demonstrated that trabectedin, in addition to direct growth inhibition, has additional immunomodulatory effects, which exerts significant effects on the tumor microenvironment (see above) that may help to explain this phenomenon which commonly becomes apparent after several cycles of treatment. Thus, any decision to stop treatment with trabectedin should always be carefully evaluated by the clinician. Treatment duration with trabectedin as an important factor for long-term outcomes was reported in the French expanded access program [107]. In that study among the 56 patients who were not progressing after 6 cycles, the subgroup of 40 patients treated with seven or more cycles had a significantly longer median PFS (10.5 months versus 5.3 months, *p* = 0.001) that translated into a more than doubling of the median OS (33.4 versus 13.9 months, *p* = 0.009) as compared to patients who stopped after six initial cycles. The results of a large retrospective analysis of trabectedin in 885 patients with advanced STS further reinforce these observations reporting that patients with nonprogressive disease who received trabectedin until disease progression obtained a statistically significant superior median PFS (11.0 versus 7.2 months, *p* < 0.003) and median OS (25.1 versus 16.9 months, *p* = 0.001) compared to those who stopped the trabectedin treatment earlier [108]. Given that the retrospective nature of the study implies potential bias, these results reinforced the rationale for performing a prospective, randomized T-DIS study (ClinicalTrials.gov Identifier: NCT01303094) within the French Sarcoma Group to compare interruption versus continuation of trabectedin in responding patients after six cycles of treatment in 178 pretreated patients with advanced STS. The final result of T-DIS trial was recently reported at the 39th European Society for Medical Oncology (ESMO) congress and strongly supported continued long-term therapy with trabectedin in responding patients until intolerance/progression, since continuation of trabectedin beyond six cycles was well-tolerated and associated with a statistically significant improvement of median PFS (continuous treatment 7.2 months versus treatment interruption 4.0 months; *p* = 0.03) [109].

Noncumulative myelosuppression, with reversible neutropenia as the predominant component, and transient transaminase increases are the most common laboratory abnormalities seen with trabectedin, both of which are associated with a low incidence of relevant clinical consequences [110]. Premedication with 20 mg of dexamethasone i.v. 30 minutes prior to trabectedin provides hepatoprotective effects beyond its antiemetic effect [52, 110, 111]. In agreement with the safety profile of trabectedin the overall incidence and severity of these events decrease in frequency over cycles demonstrating no evidence of cumulative toxicity [110, 112, 113]. Common trabectedin-related adverse events reported in at least 20% of patients are nausea, fatigue, and vomiting, whereas only 3.7% and 5.7% of patients had alopecia and mucositis/stomatitis, respectively [110]. The safety profile of trabectedin, with a lack of end-organ cumulative toxic effects, compares favorably with those of other treatments for STS, especially compared to doxorubicin-induced cumulative cardiotoxicity which prevents prolonged treatment and retreatments in most cases [114]; renal toxicity and dose-limiting neutropenia have been largely associated with ifosfamide [115], and a high rate of severe myelosuppression and pulmonary toxicity are reported after the treatment with the combination of gemcitabine plus docetaxel [42, 45]. In contrast to this, trabectedin has an acceptable safety profile even in patients who remained on therapy for prolonged periods of time (i.e., up to 59 cycles), which potentially facilitates long-term treatment until disease progression or discontinuation for other reasons [57, 110].

### 3.2. Trabectedin in Uterine Leiomyosarcoma

**Figure 1:**
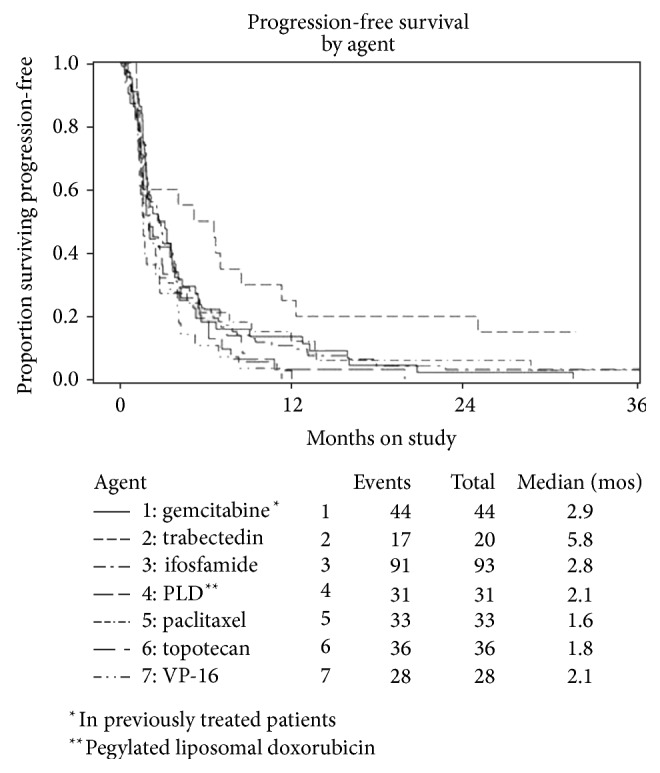
Kaplan–Meier plots demonstrating progression-free survival (PFS) for the 20 patients in the study population (GOG 87M) compared to other single agent studies in the GOG protocol 87 series studying cytotoxic agents. Reprinted from [49], with permission from Elsevier.

The GOG in the USA has conducted a prospective phase II study of trabectedin in chemotherapy-naïve patients with measurable advanced, persistent, or recurrent U-LMS with documented disease progression who were not previously exposed to chemotherapy and/or biological therapy [49]. Overall, 20 patients were enrolled and treated with trabectedin 1.5 mg/m^2^ as a 24-hour infusion q3w. Two patients achieved partial responses (10%, 95% CI: 1.2%–31.7%) with response durations of 3.3 months and 5.7 months, respectively (Table 2). Disease stabilization was reported in an additional 10 patients (50%) giving a DCR of 60%. The median PFS was 5.8 months, while the median OS was 26.1+ months. The median PFS obtained with trabectedin was compared to that obtained with other single agents in the GOG 87 series of phase II studies among chemotherapy-naïve patients. A clinically relevant delay in progression associated with the use of trabectedin (median PFS = 5.8 moths) was the longest achieved in those GOG protocol series (Figure 1). Importantly, more than half the patients remained progression-free and without any evidence of treatment-ending toxicity for more than 10 cycles (>6 months). Regarding safety issues, the most common grade 3/4 was noncumulative neutropenia (16/20 patients) associated with infection in one patient. Even though trabectedin demonstrated modest response rate in this trial, the authors conclude that PFS rather than ORR would have been a better metric to assess activity of this drug in U-LMS.

The preclinical results prompted two phase I, dose-finding trials of trabectedin and doxorubicin in patients with recurrent or persistent STS to determine the dose of trabectedin plus doxorubicin with granulocyte colony-stimulating factor (G-CSF) support [116, 117]. The MTD of trabectedin and doxorubicin given in 3-week cycles was doxorubicin 60 mg/m^2^ immediately followed by trabectedin 1.1 mg/m^2^ given as a 3 h i.v. infusion. Results from a phase I study provided the rationale to evaluate the combination of trabectedin and doxorubicin for patients with advanced LMS. The French Sarcoma Group have recently presented the results of a phase II single-arm study of trabectedin in combination with doxorubicin as first-line treatment of locally advanced and/or metastatic leiomyosarcoma of the uterus (U-LMS) or soft tissue origin (ST-LMS) [50]. The patients were stratified by primary tumor location, so the U-LMS and ST-LMS cohorts were each considered to be independent phase II studies. A total of 108 patients were treated, 47 patients in the U-LMS cohort and 61 in the ST-LMS cohort. Patients received doxorubicin 60 mg/m^2^ on day 1, followed by 3-hour intravenous infusion with trabectedin 1.1 mg/m^2^ every three weeks for a maximum of six cycles of treatment. In the U-LMS group, 28 out of 47 evaluable patients achieved a partial response (59.6%) (Table 2). A further 13 patients (27.7%) had SD yielding a DCR of 87.2%. Median PFS was 8.2 months with 87% (95% CI: 75–94) of patients remaining progression-free at 3 months. With a median follow-up of 14.5 months, median OS was 20.2 months in the uterine cohort. These efficacy results compare very favorably with outcomes reported in other studies with combination regimens in the first-line treatment of U-LMS [32, 43]. The safety profile of trabectedin plus doxorubicin was similar in pattern with phase I dose-ranging study reporting neutropenia (45%), ALT increase (14%), and thrombocytopenia (17%) as the most common grade 3/4 treatment-emergent adverse events (AEs) [116]. This safety profile was considered potentially more acceptable than that of the doxorubicin plus ifosfamide and gemcitabine plus docetaxel combination given in the first-line setting [39, 43]. Overall, the findings in these homogeneous cohorts of patients consistently confirm that trabectedin in combination with doxorubicin as first-line chemotherapy is an active treatment that provides clinically meaningful benefits to patients with U-LMS with predicted and manageable toxicity.

In addition to the ET-743-STS-201 study, a number of other phase II clinical trials with trabectedin have enrolled pretreated patients with advanced U-LMS [53–57]. A retrospective pooled analysis was performed using data on 62 patients derived from five completed phase II trials with the aim to provide an overview of the efficacy and the safety of trabectedin in U-LMS [51] (Table 3). Most of the patients (91.9%) had been pretreated with a median of 2 prior chemotherapy regimens (range: 0–6; five patients were chemotherapy-naïve), 98.4% had undergone prior surgery, and 48.4% had prior radiotherapy. In all studies trabectedin 1.5 mg/m^2^ was given as a 24-hour i.v. infusion q3w. Across trials, patients received a median of 3 cycles per patient, reaching up to 38 cycles with no signs of cumulative toxicities. According to investigators' assessment partial responses were observed in 11 patients (17.7%; 15% ≥6 months) and SD in 20 patients (32.3%; 13% ≥6 months) for a DCR of 53.2% (Table 2). For the entire patient population median PFS was 2.5 months (95% CI: 1.7–4.2) with 46.4% (95% CI: 33.7%–59.1%) and 30.8% (95% CI: 19.0%–42.7%) progression-free at 3 and 6 months, respectively. Median OS was 12.1 months (95% CI: 7.5–14.0), with 12- and 24-month OS rates of 51.6% (CI 95%: 39.2–64.1) and 20.3% (CI 95%: 10.1–30.4), respectively. The most common patient grade 3/4 adverse events were noncumulative neutropenia (41.9%) and transient asymptomatic transaminase increases of ALT and AST observed in 43.5% and 30.6% of patients, respectively, without symptoms of hepatic failure. Thus, the results of phase II studies confirm trabectedin as an efficacious single agent for the treatment of advanced U-LMS with the safety profile that favorably compares with those of other active drugs, including those who remained on therapy for prolonged periods of time [51].

An Italian phase II randomized, noncomparative, cross-over TAUL trial (EudraCT number 2009-016017-24) is currently assessing the activity of trabectedin and gemcitabine plus docetaxel in metastatic or locally relapsed uterine LMS pretreated with conventional chemotherapy.

The aforementioned results correspond to clinical studies which, by nature, are restrictive in the characteristics of the patients included. In the absence of large randomized studies, observational studies performed in clinical practice, although not as methodologically rigorous, can provide useful insights into the real-world efficacy, toxicity, and management of patients treated with trabectedin and show how results from clinical trials may translate in a “real-world” setting. Sanfilippo et al. carried out a retrospective analysis of all patients with advanced U-LMS treated with trabectedin from 2000 to 2010 at two European sarcoma reference centers (Istituto Nazionale Tumori, Milan, and Royal Marsden Hospital, London) [52]. Overall, 66 patients with metastatic U-LMS who had failed a median of three prior cytotoxic lines including anthracyclines with or without ifosfamide (100% of patients) and gemcitabine with or without docetaxel (87% of patients) were included in the analysis. Eleven patients achieved a partial response (16%) and an additional 23 (35%) achieved SD (three of them showing minor tumor shrinkage) for a DCR of 51%. Interestingly, two patients achieved a delayed response to treatment, showing a partial response (after a decrease in tumor density) and a minor response after 14 and 10 cycles, respectively. After a median follow-up of 22 months, the median PFS was 3.3 months (95% CI: 2.7–5) with 53% and 33% of patients progression-free at 3 and 6 months, respectively. The median OS was 14.4 months (95% CI: 8–20). Thus, the efficacy outcomes of this study in an unselected patient population representative of routine clinical practice were consistent with those seen in more selective populations enrolled in clinical trials.

## 4. Conclusions

Standard treatment for early U-LMS is hysterectomy with BSO. Adjuvant radiotherapy and chemotherapy are not administered since they do not result in a survival benefit. Treatment outcomes in U-LMS are far from being satisfactory, especially in patients with inoperable, locally advanced, and/or metastatic disease. Many patients with recurrent LMS receive multiple lines of therapy but the optimal sequencing of these drugs into the treatment algorithm for U-LMS has not been well defined. Available data from phase II studies and observational studies have demonstrated that trabectedin has significant activity in patients with advanced U-LMS with a high DCR ranging from 51% to 60% and an acceptable safety profile. In addition, trabectedin results in 30% PFS rate at 6 months with 12-month OS rate of more than 50% in pretreated patients with U-LMS. Taken together, the response rate, PFS, and OS with trabectedin are comparable with published outcomes on other single agents (doxorubicin, ifosfamide, and gemcitabine) in this indication [118].

Regarding safety, current treatment options for patients with U-LMS are frequently guided by safety considerations and convenience. Many of the currently available chemotherapeutics or combinations used in U-LMS are associated with cumulative, duration limiting, or irreversible toxicities that may jeopardize future long-term interventions. The safety profile of trabectedin compares favorably with that of other active drugs used in U-LMS, including those who remained on therapy for prolonged periods of time, as it allows patients to benefit from a longer-term treatment, with the potential for longer disease control.

Finally, the results from the GOG and the French Sarcoma Group phase II studies show very promising results of trabectedin as first-line therapy either as single agent or in combination with doxorubicin. Particularly, the findings of a phase II study of trabectedin in combination with doxorubicin demonstrated the feasibility of this combination reporting an encouraging synergistic and clinically meaningful response in patients with U-LMS with an acceptable and predictable tolerability profile. Noteworthily trabectedin plus doxorubicin yielded numerically higher response rate and superior survival compared with historical results of the two most active combination regimens (gemcitabine plus docetaxel and doxorubicin with or without ifosfamide) for advanced U-LMS. However, the difficulty lies in knowing where these regimens fit into the treatment algorithm for U-LMS given that there are no randomized comparisons of these regimens. Therefore, potential future combination of trabectedin with additional active agents should be further explored in patients with U-LMS as first- or second-line treatment.

## Figures and Tables

**Table 1:** Summary of efficacy results of active chemotherapy regimens in uterine leiomyosarcoma (for trabectedin data see Table 2).

Drug(s)	Evaluable patients (*n*)	Trial design	Prior regimen(s)	ORR (%)	SD (%)	Median PFS (months)	Median OS (months)
Doxorubicin [35]	Uterine STS (72 with U-LMS)	Randomized phase III	0	16.3 (all) 25 (U-LMS)	NR	NR	12.1

Doxorubicin [34]	Uterine STS (38 with U-LMS)	Randomized phase III	0	19 (all) 13 (U-LMS)	54.0 (all) 70.0 (U-LMS)	5.1	NR

Ifosfamide [36]	35 U-LMS	Phase II	0	17.2	28.6	NR	6.0

Doxorubicin + ifosfamide [32]	34 U-LMS	Phase II	0	30.3	51.7	NR	9.6

Doxorubicin + ifosfamide [37]	Uterine STS (25 with U-LMS)	Phase I/II	0	49.0 (all) 48.0 (U-LMS)	30.0 (all)	NR	30.5 (all)

Gemcitabine [40]	42 U-LMS	Phase II	0-1	20.5	15.9	NR	NR

Gemcitabine + docetaxel [43]	39 U-LMS	Phase II	0	35.8	26.2	4.4	16.0+

Gemcitabine + docetaxel [42]	48 U-LMS	Phase II	1	27.0	50	6.7+	14.7

Gemcitabine + docetaxel [44]	LMS (29 with U-LMS)	Phase II	0–2	53.0	20.6	5.6	17.9

Gemcitabine + docetaxel [45]	Advanced STS (38 with U-LMS)	Randomized phase II	0–3	16.0 (all) 17.0 (U-LMS)	NR	6.2	17.9

Gemcitabine + docetaxel [47]	Advanced LMS (46 with U-LMS)	Randomized phase II	1	5.0 (LMS) 24.0 (U-LMS)	NR	3.4 (LMS) 4.7 (U-LMS)	13.0 (LMS) 23.0 (U-LMS)

NR: not reported; ORR: objective response rate; OS: overall survival; PFS: progression-free survival; SD: stable disease; STS: soft tissue sarcoma; U-LMS: uterine leiomyosarcoma.

**Table 2:** Summary of efficacy results of trabectedin in advanced uterine leiomyosarcoma.

Study	Regimen	Evaluable patients (*n*)	Prior regimens Median (range)	ORR	DCR	Median PFS (months)	3-month PFS	6-month PFS	Median OS (months)	12-month OS	24-month OS
Phase II GOG study [49]	Trabectedin	20	0	10%	60%	5.8	NR	NR	26.1+	NR	NR
Phase II LMS-02 study [50]	Trabectedin/doxorubicin	47	0	59.6%	87.2%	8.2	87%	NR	20.2	NR	NR
Pooled analysis [51]	Trabectedin	62	2 (0–6)	17.7%	53.2%	2.5	46.4%	30.8%	12.1	51.6%	20.3%
Retrospective analysis [52]	Trabectedin	66	3 (1–5)	16%	51%	3.3	53%	33%	14.4	NR	NR

DCR: disease control rate; GOG: Gynecologic Oncology Group; NR: not reported; ORR: objective response rate; OS: overall survival; PFS: progression-free survival; U-LMS: uterine leiomyosarcoma.

**Table 3:** Patients included in pooled analysis of five phase II studies.

Phase II studies	Reference	Evaluable patients (*n*) Total *n* = 62	1st line therapy
ET-B-005	Le Cesne et al. [53]	16	No
ET-B-008	Yovine et al. [54]	7	No
ET-B-016	Garcia-Carbonero et al. [55]	6	Yes
ET-B-017	Garcia-Carbonero et al. [56]	3	No
ET743-STS-201	Demetri et al. [57]	30	No
